# Amorphous Calcium Carbonate Precipitation by Cellular Biomineralization in Mantle Cell Cultures of *Pinctada fucata*

**DOI:** 10.1371/journal.pone.0113150

**Published:** 2014-11-18

**Authors:** Liang Xiang, Wei Kong, Jingtan Su, Jian Liang, Guiyou Zhang, Liping Xie, Rongqing Zhang

**Affiliations:** 1 Institute of Marine Biotechnology, School of Life Sciences, Tsinghua University, Beijing, China; 2 Laboratory for Reproductive Health, Institute of Biomedicine and Biotechnology, Shenzhen Institutes of Advanced Technology, Chinese Academy of Sciences, Shenzhen, China; 3 Protein Science Laboratory of the Ministry of Education, Tsinghua University, Beijing, China; University of Iowa, United States of America

## Abstract

The growth of molluscan shell crystals is generally thought to be initiated from the extrapallial fluid by matrix proteins, however, the cellular mechanisms of shell formation pathway remain unknown. Here, we first report amorphous calcium carbonate (ACC) precipitation by cellular biomineralization in primary mantle cell cultures of *Pinctada fucata*. Through real-time PCR and western blot analyses, we demonstrate that mantle cells retain the ability to synthesize and secrete ACCBP, Pif80 and nacrein *in vitro*. In addition, the cells also maintained high levels of alkaline phosphatase and carbonic anhydrase activity, enzymes responsible for shell formation. On the basis of polarized light microscopy and scanning electron microscopy, we observed intracellular crystals production by mantle cells *in vitro*. Fourier transform infrared spectroscopy and X-ray diffraction analyses revealed the crystals to be ACC, and *de novo* biomineralization was confirmed by following the incorporation of Sr into calcium carbonate. Our results demonstrate the ability of mantle cells to perform fundamental biomineralization processes via amorphous calcium carbonate, and these cells may be directly involved in pearl oyster shell formation.

## Introduction

Biomineralization refers to the process of hard tissue formation by organisms and has been characterized as a highly controlled and functional process [1]. The pearl oyster *Pinctada fucata* is one of the best-studied species with regard to biomineralization due to its intriguing shell microstructure, which consists of inner aragonitic nacreous and outer calcitic prismatic layers; in addition, *Pinctada fucata* is of economic importance to the pearl industry [2]. The process of aragonitic nacreous layer formation is a promising model for the development of biomaterials for a wide variety of applications in such varied fields as nanotechnology, biomedical engineering, tissue regeneration and crystal growth [3]. Indeed, an in-depth understanding of this complex process can lead to new ideas for synthetic crystallization processes of interest to materials science.

Amorphous calcium carbonate (ACC) is the precursor phase of both aragonite and calcite [4]: ACC destined to be transformed into aragonite has a nascent aragonite-like order, whereas ACC destined to be transformed into calcite has a nascent calcite-like order [5]. Many studies have shown that ACC plays a crucial role in the formation of mineralized tissues [6]–[11]. In nature, organisms can produce stable spherical ACC particles, and the colloidal nanoparticles participate as transient intermediates in the formation of crystalline aragonite or calcite, such as in mollusc shells and sea urchins. Beniash *et al.* have shown that ACC is present in the sea urchin larval spicule, which was the first documentation of the biological transformation of ACC into calcite [8]. During the development of the freshwater snail *Biomphalaria glabrata*, the first stage is an ACC phase in which the structure of aragonite is already preformed [9]. Weiss *et al.* and Miyazaki *et al.* also reported the existence of ACC in the larval shells of the marine bivalves *Mercenaria mercenaria*, *Crassostrea gigas* and *Pinctada fucata*, showing that ACC was subsequently transformed into aragonite [10]–[11]. ACC fulfills an important function in mollusc larval shell formation, and ACC has also been reported in the adult shell [6]; however, the mechanism of the extracellular or intracellular transformation of ACC into aragonite remains unclear.

The mantle is responsible for shell formation and secretes a matrix complex that includes proteins, polysaccharides and lipids. It has been suggested that the insoluble proteins provide the framework and mechanical properties of the shell, whereas the soluble proteins are involved in crystal nucleation and growth, thereby controlling calcium carbonate polymorphs [12]–[13]. The outer calcite prismatic layer is always related to the proteins secreted from the outer epithelial cells of the edge of the mantle, whereas the inner aragonite nacreous layer is related to the proteins of the pallial region [14]–[16]. However, little is known about the cellular aspects of molluscan shell formation, though a knowledge of this process is essential for revealing the cellular mechanisms of shell biomineralization. As demonstrated by the progress achieved by using *in vitro* models of bone and coral mineralization, long-term primary cell cultures can provide innovative tools to investigate mineralization at the cellular level [17]–[20]. Accordingly, the nature of mantle cells involved and the mechanisms of their cooperation in the regulation of mineralization can be explored using these models. Similarly, insight into shell formation may be obtained from mantle cell cultures [21].

However, the development of cell cultures from marine invertebrates has been slow when compared with the cell cultures from vertebrates and insects. Although no immortal marine invertebrate cell lines have been reported to date [22], primary cell cultures derived from marine invertebrates have been used to investigate biomineralization mechanisms at the cellular level [17], [21], with mantle cells in primary culture surviving for periods ranging from one to two months [21], [23]. The deposition of calcium carbonate crystals was firstly reported in mantle tissue cultures from the pearl oyster *Pinctada fucata* using polarized microscopy [24]. In addition, EDS analysis allowed to the determination of the CaCO_3_ nature of the deposits and their biogenic origin, and the expression and secretion of matrix proteins have also been detected in mantle explant cultures [21]. However, calcium carbonate polymorph deposited in mantle tissue culture and the cellular mechanisms of crystal formation remain unclear. Although haemocytes are thought to be directly involved in shell repair by storing intracellular calcium carbonate crystals and delivering crystals to the mineralization front [25], their contribution to normal shell formation is still under debate. Mantle cells are known to play central roles in shell and pearl formation. Considering the remarkable nacre structure and the contribution of the mantle cells to shell formation, the utilization of cell biological approaches is essential for further detailed analyses of shell formation mechanisms. Furthermore, these cells maintain cell-to-cell contacts in multicellular culture, may preserve the viability and functionality of mantle cells and may delay cellular aging and death, thus allowing *in vitro* biomineralization [22]. Hence, our focus is to identify the mechanism of aragonite nacreous layer formation by cellular biomineralization *in vitro*.

We have had previous success in establishing long-term primary cell cultures from the mantle tissue of the pearl oyster *Pinctada fucata* [21], [23]. In the present study, we extended the use of such primary cell cultures to produce intracellular ACC for preliminary evidence. The activities of alkaline phosphatase and carbonic anhydrase with a focus on their function in shell formation were examined *in vitro*. Moreover, the expression and secretion of matrix proteins ACCBP, Pif80 and nacrein during cell culture were evaluated. Our investigation attempts to determine the cellular functions of the mantle in nacre formation *in vitro*.

## Material and Methods

### Cell cultures

Cell cultures were prepared as described previously with minor modifications [21]. Briefly, small fragments of the pallial mantle were excised from *Pinctada fucata* and incubated for 20 min with gentle shaking in molluscan balanced salt solution(MBSS) supplemented with 0.5 mg/ml streptomycin, 500 IU/ml penicillin, 100 IU/ml gentamicin, and 2 µg/ml nystatin. After several rinses in D-MBSS(MBSS without Ca^2+^ and Mg^2+^), the fragments were then transferred to Petri culture dishes (Greiner Bio-one, Germany) containing 0.5 ml of Pf-CM2.5 medium supplemented with 10% fetal bovine serum (Hyclone, USA). The explants were cultured at 24°C without CO_2_. After incubation for 1 day, culture medium was added to a total volume of 1 ml and half was changed every 3 days. The Pf-CM2.5 medium was prepared as follows: equal volumes of 2× Leibovitz-15 and 2× Medium 199 (Gibco, USA) were mixed, and 170.6 mM NaCl, 40 µg/ml ascorbic acid, 128.9 µg/ml taurine, 10 µg/ml ATP, 400 µg/ml lactalbumin hydrolysate, 3.57 mg/ml HEPES, 100 IU/ml penicillin, 100 µg/ml streptomycin, 50 µg/ml kanamycin and 1 µg/ml nystatin were added. This medium contained 2.5 mM Ca^2+^ and 2.5 mM Mg^2+^, with a pH of 7.0. The cellular metabolic activity was measured with the MTT reduction assay using 5×10^4^ cells/well in 96-well plates as described previously [23]. We repeated the cell culture experiments for three times independently.

### Alkaline phosphatase and carbonic anhydrase activities

The cells were washed twice with D-MBSS and sonicated for 30 s for the detection of alkaline phosphatase (ALP) and carbonic anhydrase (CA). The cellular protein content was measured using the bicinchoninic acid assay (Pierce, Sigma), with bovine serum albumine (BSA) as the standard. The ALP activity was measured as described in Sud *et al.* [26]. The cell lysates were incubated in 0.2 M bicarbonate buffer (pH = 10), 4 mM MgCl_2_ and 2 mM p-nitrophenolphosphate at 37°C for 1 h. The reaction was stopped by adding 1 M NaOH, and the absorbance at 405 nm was measured. We measured the CA activity as described by Antonino *et al.* [27], with some modifications. The reaction was initiated by mixing the substrate p-nitrophenyl acetate (66 µl, 30 mM) (Sigma, USA) with a solution of Tris–HCl pH 7.4 (134 µl, 46 mM), containing the cell lysate (10 µg/µl). The reaction was proceeded out at 25°C for 30 min and the absorbance at 405 nm was measured. The enzyme activities were normalized to the cell protein content for analysis.

### Microscopy imaging

For detecting extracellular or intracellular calcium ions, cells from a 25-day old culture were incubated in Pf-CM2.5 containing 50 µM Calcein (Sigma) or 8 µM Fluo-3 AM (Sigma) at 24°C for 1 h. After washing three times with Pf-CM2.5, the cells were examined using a fluorescence microscope (Leica, DMIRB). Observations of the 25-day-old cells were performed using polarized microscope (Leica, DMIRB) for detecting the intracellular calcium carbonate crystals. For scanning electron microscopy with back-scattered electron (SEM-BSE) and energy-dispersive X-ray spectrometry (EDS), the cells were grown on a cover glass placed in a Petri culture dish containing Pf-CM2.5, Pf-C10M50 or Pf-S10M50 with 10% fetal bovine serum. The Pf-C10M50 and Pf-S10M50 medium were prepared in a similar way as Pf-CM2.5 but contained 50 mM Mg^2+^ with 10 mM Ca^2+^ and 10 mM Sr^2+^ plus 2.5 mM Ca^2+^, respectively. These cultures were fixed for 15 min with 4% paraformaldehyde. The samples were gently washed with distilled water, followed by dehydration in an ascending ethanol series (50–100%) and critical point drying with liquid CO_2_. All the samples were then coated with carbon and observed using an FEI Quanta 200 FEG scanning electron microscope.

### Real-time PCR analysis

The mantle cells were collected and washed twice with D-MBSS. The total RNA of the cells was extracted using the TRIzol RNA Isolation Reagent (Life Technologies), according to the manufacturer's instruction; cDNA was prepared by reverse transcription-PCR of total the RNA with PrimeScript RT Reagent Kit (Takara) following the manufacturer's instructions. The real-time quantitative PCR was conducted with the Mx3000P RT-PCR system (Stratagene) using the SYBR Premix Ex Taq II kit (Takara). The cycle conditions were as follows: 1 cycle of 95°C for 30 s and 40 cycles of 95°C for 5 s and 60°C for 20 s. The dissociation curves were analyzed to determine the purity of the product and the specificity of the amplification. Three genes encoding matrix proteins (nacrein, Pif80 and ACCBP) were targeted, and the housekeeping gene actin was selected as the reference for the calculation of the relative expression levels of the genes. The primer sequences for each gene are given in Table S1.

### Western Blot Analysis

An expression plasmid was constructed by inserting the Pif80 gene into the PET28a vector. Recombinant Pif80 with a His_6_ tag at the C terminus was overexpressed with 1 mM IPTG in *Escherichia coli* (Transsetta DE3) at 37°C for 4 h. The cells were homogenized and the protein was purified using a His-trap FF crude column attached to an ATKA purifier (GE Healthcare) according to the manufacturer's instructions. The eluted fractions containing purified recombinant Pif80, as detected by SDS-PAGE were desalted using a HisTrap desalting column (GE Healthcare). Polyclonal antibodies against Pif80 were raised in a New Zealand rabbit following standard immunization procedures; the antibodies were affinity-purified using an antibody purification kit (ZKCY-BIO) according to the manufacturer's instructions. Antibodies against nacrein and ACCBP were prepared by Gong *et al.* and Ma *et al.*, respectively [21],[28]. Protein samples, including the medium of the mantle tissue cell culture and uncultured medium, were separated by SDS-PAGE and transferred to a PVDF membrane (Millipore). The membrane was treated with a 1∶2000 dilution of antibody against ACCBP, 1∶1000 dilution of antibody against Pif80, 1∶500 dilution of antibody against nacrein, and 1∶1000 dilution HRP-conjugated goat anti-rabbit antibody (Calbiochem), respectively. The membrane was incubated with Luminata Crescendo Western HRP Substrate (Millipore) and exposed to X-ray film for 1 min. Control experiments were performed without the incubation of the primary antibody.

### *In Vitro* Calcium Carbonate Crystallization Assay

A saturated calcium bicarbonate solution was prepared following the method of Xu *et al.* [29], with some modifications. CO_2_ gas was bubbled through a mixture of calcium carbonate and Milli-Q deionized water for 8 h, and the excess solid CaCO_3_ was removed by filtration. Crystallization experiments were conducted by adding samples to the freshly prepared crystallization solution on a siliconized cover glass. After 24 h, the crystallization solution was removed, and the crystals were characterized using an FEI Quanta 200 scanning electron microscope and Raman spectroscopy, with recording at an excitation wavelength of 514 nm. The spectra were scanned three times for 20 s in the range of 100–1400 cm^−1^ using a Renishaw RM2000 spectrometer.

### Characterization Of Caco_3_

The mantle cells were cultured in Pf-C10M50 for 25 days; 10 dishes of cell culture were combined and centrifuged. The pellet was rinsed with Milli-Q deionized water and stored in ethanol for analysis. The ethanol was removed and we prepare the cell powder by critical point drying with liquid CO_2_ before analysis, Fourier transform infrared spectroscopy (FTIR) spectra were collected in the range of 200–2000 cm^−1^ using a Nicolet 560 Fourier transform infrared spectrometer. The X-ray diffraction (XRD) data were collected using a D/max 2500 X-ray diffractometer, with Cu Kα radiation at 50 kV and 200 mA. The samples were scanned between 2θ values of 10 and 90° at a scan speed of 1.2°/min. Synthetic ACC, according to the method of Koga *et al.*, was used as a reference for further XRD analysis [30]. Equimolar sodium carbonate (0.1 M) and calcium chloride (0.1 M) solutions were mixed with vigorous stirring at 0–4°C. Sodium hydroxide (1 M) was added to obtain mixed solutions within pH 11.2–11.4. Then the precipitated colloidal phase was centrifuged for 10 min at 2000×g and 4°C to separate the ACC.

### Statistical Analysis

For the analysis of the expression data, we standardized the 2^−ΔΔCt^ of each gene by taking the 5th day as the zero point. All the data in this study were compared using one-way analysis of variance (ANOVA) and post-hoc comparisons using Duncan's Multiple Range Test with the software SPSS 20.0. All tests were performed at a significance level of 5%.

## Results

### Cell Culture Characterization

**Figure 1:**
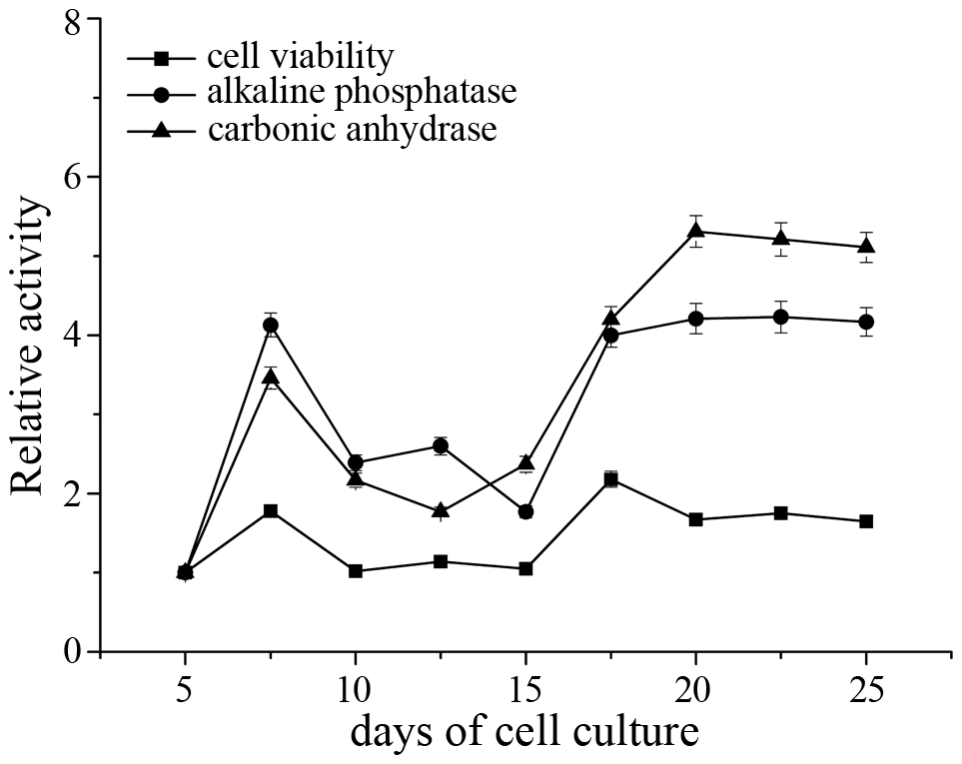
The relativity cell viability, alkaline phosphatase, and carbonic anhydrase activities during cell culture. The activity of each treatment at 5 days was normalized to 1.

**Figure 2:**
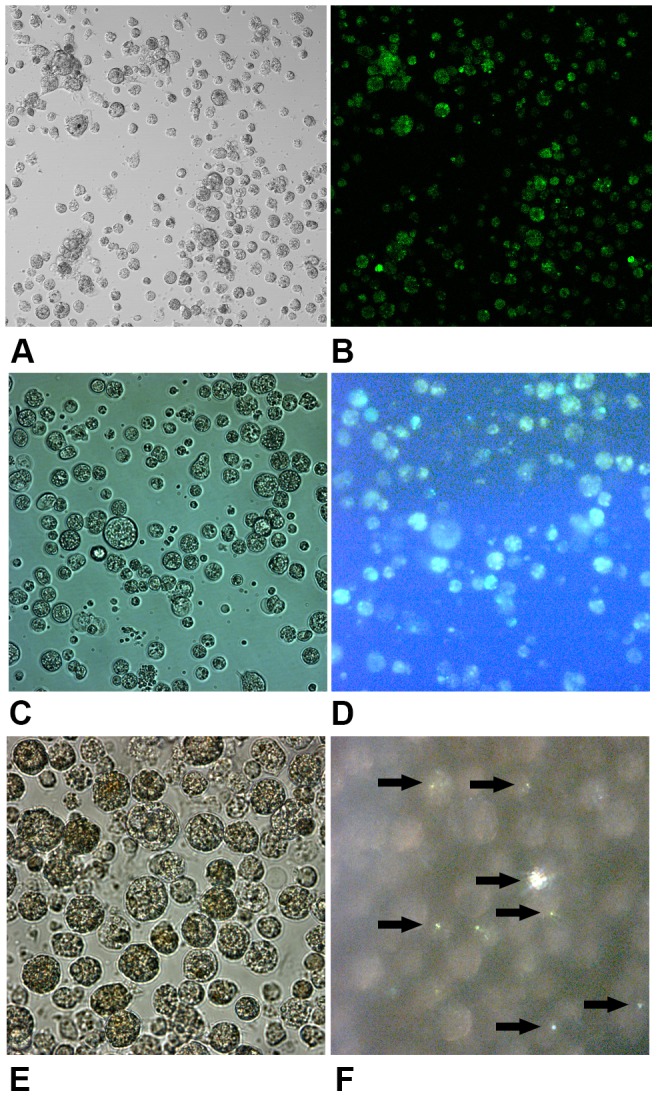
Micrographs of the mantle cells at 25 days. (A) and (B) Cells labelled with fluo-3 AM (magnification 200x); (C) and (D) cells labelled with calcein (magnification 400x); (E) and (F) cells containing crystals (magnification 630x), as indicated with arrows. White light in (A), (C) and (E); fluorescent light in (B) and (D); polarized light in (F).

The primary cell cultures displayed high degrees of cellular metabolic activity over a period of 25 days (Figure 1). There was an initial increase in cellular metabolic activity from 5 to 7.5 days and a significant decrease from 10 to 15 days; the cellular metabolic activity increased suddenly at 17.5 days, and showed high levels from 17.5 to 25 days. The variability of the alkaline phosphatase (ALP) and carbonic anhydrase (CA) activities was similar to the cellular metabolic activity during cell culture (Figure 1). The ALP and CA activities peaked at 7.5 days of cell culture, decreased significantly from 10 to 15 days and then increased gradually from 15 to 20 days and remained at high levels from 20 to 25 days (Figure 1). The intracellular and extracellular calcium ion distribution in the mantle cells were observed using the fluorescent dyes fluo-3 AM and calcein, respectively (Figure 2B, D), and some of the cells contained an abundance of calcium ions. Further evidence that mineral crystals were present in the mantle cells and information on the nature of the mineral phase was obtained by the observation of cells using polarized light microscopy. A weak birefringence indicative of crystallization was observed in the cells (Figure 2F arrows), whereas no birefringence was observed before 10 days of cell culture (data not shown).

### Synthesis And Secretion Of Matrix Proteins By Mantle Cells

**Figure 3:**
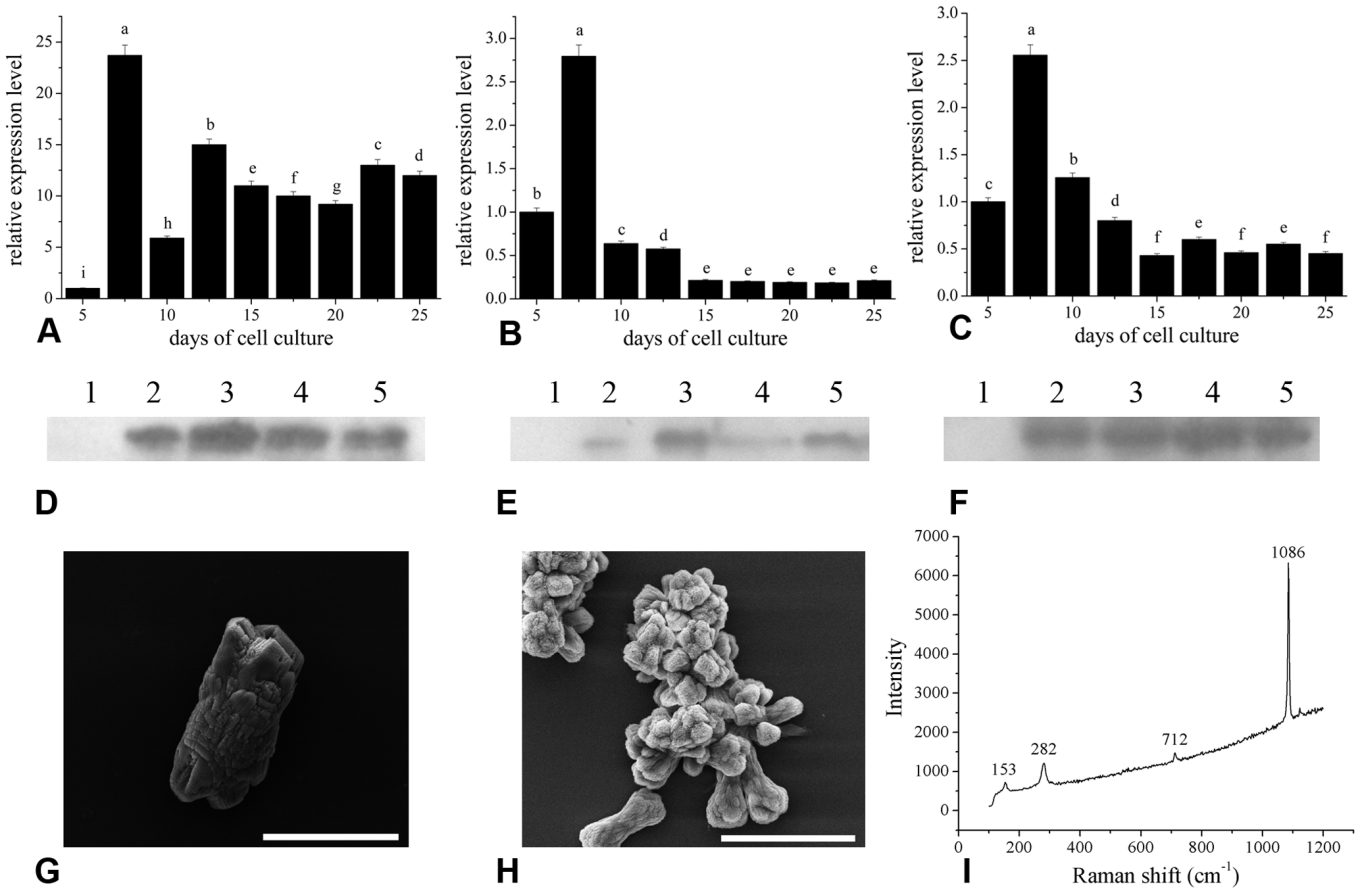
The expression and secretion of shell matrix proteins by mantle cells. (A), (B) and (C) The relative expression levels of ACCBP, nacrein and Pif80 during cell culture, respectively. (D), (E) and (F) Western blot analysis of secreted proteins using antibodies against ACCBP, nacrein and Pif80, respectively. Uncultured medium in lane 1 as a control, cell culture medium at 10, 15, 20 and 25 days in lanes 2, 3, 4 and 5, respectively. (G) and (H) The effects of uncultured medium and medium from cell culture at 25 days on calcium carbonate crystallization, respectively. The aggregation and morphology of calcites were affected by the medium from cell culture. (I) Raman spectra of the crystals formed in (G) and (H); the characteristic peaks of calcite are at 153, 282, 712 and 1086 cm^−1^. Scale bar 20 µm.

The expression of matrix proteins in the mantle cells *in vitro* was examined using real-time PCR. The expression level of ACCBP increased significantly and reached a maximum at 7.5 days and then remained at a high level from 10 to 25 days (Figure 3A). The expression levels of nacrein and Pif80 showed similar patterns (Figure 3B, C), which increased significantly and reached a maximum at 7.5 days, decreased suddenly at 10 days and showed low levels from 10 to 25 days. We also detected matrix protein secretion by western blotting using antibodies against ACCBP, nacrein and Pif80. ACCBP, nacrein and Pif80 were detected in the cell culture medium from 10 to 25 days (Figure 3D, E, F). *In vitro* crystallization experiments were conducted to further explore the role of the matrix proteins found in the cell culture medium in biomineralization. In the presence of uncultured medium control, the precipitated crystals were all typical rhombohedra of calcite (Figure 3G), whereas the aggregation of acicular calcites was observed in the presence of the culture medium (Figure 3H). The addition of culture medium provoked a strong effect on the morphology of calcites, which indicated that the matrix proteins retained the ability to regulate crystal growth *in vitro*.

### Intracellular Acc Precipitation By Mantle Cells

**Figure 4:**
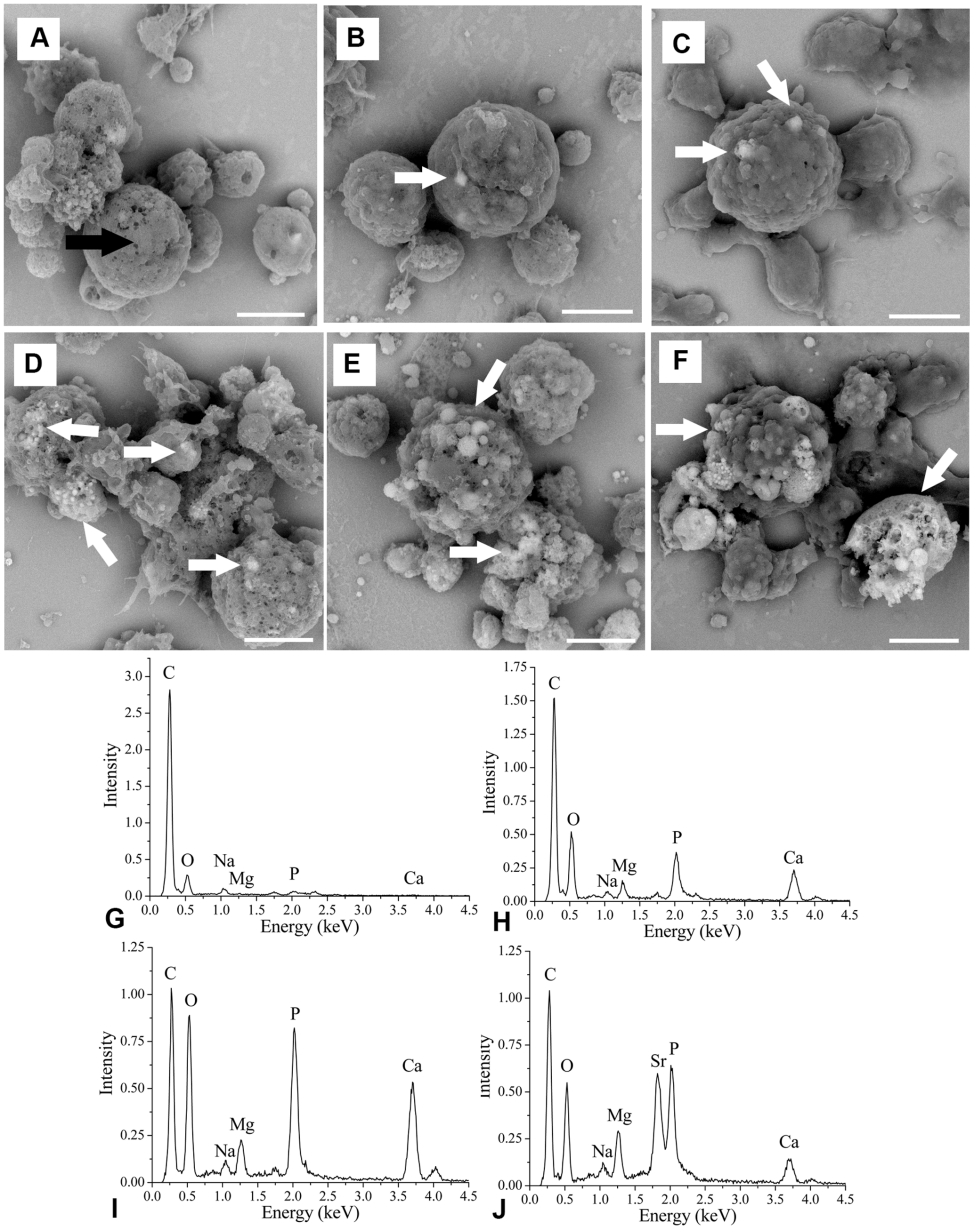
Backscatter electron imaging combined with energy dispersive X-ray spectral analysis of the mantle cells. In the Backscatter electron (BSE) mode, elements with a higher atomic number scatter more electrons (such as calcium and strontium), thus appearing lighter, whereas carbon appears black. (A), (B), (C) and (D) SEM-BSE images of cells at 10days, 15 days, 20 days and 25 days, respectively, in Pf-CM2.5. (E) and (F) SEM-BSE images of cells at 25 days in Pf-C10M50 and Pf-S10M50, respectively; the intracellular crystals are indicated with white arrows. (G) Energy dispersive X-ray spectral (EDS) of the cells in (A), the elemental composition of the cells consisted of C and O. The black arrow on the SEM image indicates the sample point of the EDS. (H) EDS of the crystals in (B), (C) and (D). The elemental composition of the crystals is C, O, Mg, P and Ca. (I) and (J) EDS of the crystals in (E) and (F), respectively. The elemental composition of the crystals is C, O, Mg, P and Ca in (E) and C, O, Mg, P, Ca and Sr in (F). The white arrows on the SEM image indicate the sample point of the EDS. Scale bars 10 µm.

**Figure 5:**
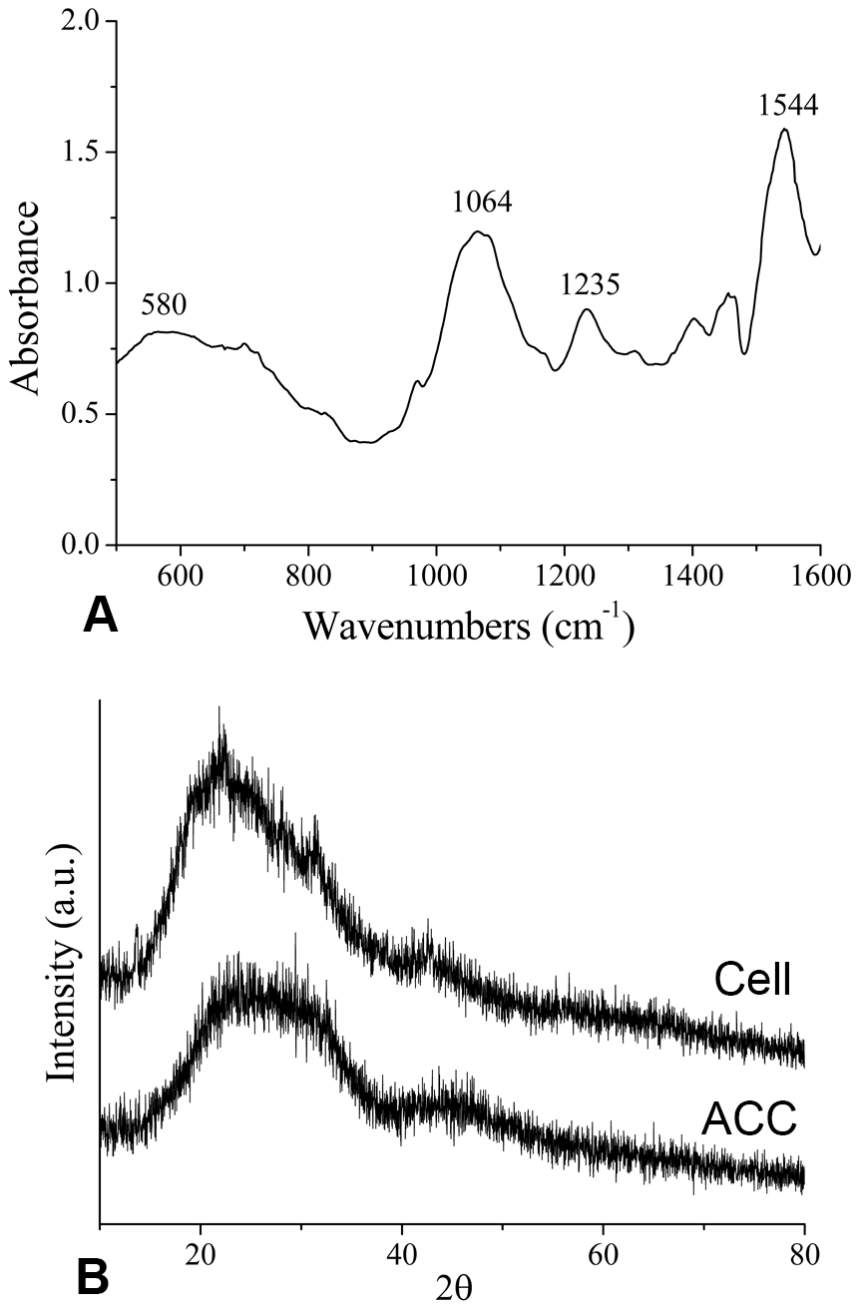
FTIR and XRD of intracellular crystals showing characteristic bands indicating the presence of ACC. (A) FTIR spectra of powdered intracellular crystals; the broad bands at 580,1064,1235 and 1544 cm^−1^ are not related to a distinct calcium carbonate polymorph (calcite or aragonite). (B) X-ray powder diffraction (XRD) pattern of intracellular crystals (Cell) and synthetic ACC (ACC); the intracellular crystals and synthetic ACC exhibit the same characteristic bands for ACC.

No deposits were observed in the mantle cells incubated in Pf-CM2.5 for 10 days (Figure 4A). In contrast, mineral deposits with irregular or spherical morphology were visible within the mantle cell from 15 to 25 days, and the number of deposits was greatly increased by the cell culture duration (Figure 4B, C, D). When the mantle cell cultures were incubated in Pf-C10M50 containing Ca^2+^ and Mg^2+^ in concentrations similar to the oyster extrapallial fluid [31], the mineral deposits became larger and were increased compared to that in Pf-CM2.5 (Figure 4E). The spot EDS analysis showed that the compositions of these deposits were similar, including significant amounts of calcium, carbon, phosphorus and oxygen, with small amounts of magnesium (Figure 4H, I); the composition of the cell was carbon and oxygen (Figure 4G). The detection of P in the calcium deposition is compatible with the presence of an ACC phase, as recently proposed by Jacob *et al.* [32]. To confirm that the calcium deposition was formed *de novo*, we measured the incorporation of Sr into the deposition. When the mantle cells were incubated in Pf-S10M50 for 25 days, many deposits were observed in the mantle cell (Figure 4F). The spot EDS analysis showed that the composition of deposits included significant amounts of strontium, carbon, oxygen, and phosphorus, with small amounts of magnesium and calcium (Figure 4J). To ascertain the polymorphs of intracellular calcium deposition, the powdered cells were analyzed by FTIR and XRD. The FTIR spectra of the mineral phases obtained from the mantle cells suggested that ACC could be present in the precipitate (Figure 5A). In the XRD diffraction profile, a broad band spanning between 20° and 40° of 2θ, which was same as synthetic ACC, was found (Figure 5B).

## Discussion

ALP is a type I phosphomonoesterase that hydrolyzes short chain esters, and CA catalyses the reversible hydration of CO_2_, accelerating bicarbonate formation; these enzymes have been related to the process of shell formation in molluscs [33],[34]. The enzyme activities first peaked at 7.5 days prior to nucleation and growth of ACC and remained at high levels after 15 days of cell culture with ACC first appearing in the mantle cell at 15 days (Figure 4B). The primary cell cultures from the pearl oyster *Pinctada fucata* displayed an increase in mineralization-related enzymes activities that was followed by ACC precipitation. ALP is a nonspecific enzyme, which has been related to the process of *in vitro* crystalline biomineralization in mammalian ossification models of bone-forming osteoblasts. In mollusks, ALP is a ubiquitous enzyme involved in biomineralization, primary cell cultures displayed a increase in ALP activity, enzyme activation was followed by precipitation of ACC. These results strongly suggest a role of ALP in the regulation of molluskan shell mineralization, It is used as a marker of early biomineralization activity. Increased ALP activity is interpreted as a prerequisite initial step, possibly related to cell-mediated secretion of structural organic matrix, which then mineralizes in a second step into the final calcified tissue product. These findings are consistent with a previous study that reported that the ALP and CA activities are linked to shell formation throughout larval development [34]. The mantle cells also showed a constant MTT response that reflected a constant cellular metabolic activity that was similar to the enzymes activities. These results confirmed the growth potential of cells *in vitro* and showed that mantle cells can be used for further cellular biomineralization studies.

The intracellular and extracellular calcium ion distributions in the mantle cells were examined by fluorescent indicators (Figure 2B, D). Some of the mantle cells are involved in sequestering and concentrating the ions to be used for mineralization, and these cells may need to take up Ca^2+^ continuously to maintain an internal calcium pool. The most widely discussed models of ion uptake for mineralization purposes involve membrane ion transporters and channels that allow the ions to enter the cell and then to follow a pathway to the site of mineralization; accordingly, our results indicated that calcium storage and utilization in the manle cells were essential for cellular biomineralization. Bright birefringence, which represents the mineral phase, was identified in the mantle cells at 25 days (Figure 2F), whereas no birefringence was observed before 10 days of cell culture. We assumed that the crystals were formed by mantle cell biomineralization, and a similar appearance was observed during abalone larvae development [9],[34]. One class of the haemocytes contained granules that were birefringent when examined by polarized light microscopy [25], and some of these granules might be calcium carbonate crystals.

It has been revealed that *in vitro* mantle tissue can synthesize matrix proteins, such as MSI7, MSI60 and nacrein [21]. We demonstrated that the expression of ACCBP, nacrein [35] and Pif80 [36] were retained in the *in vitro* mantle cells over a period of 25 days after culture initiation (Figure 3A, B, C). This expression increased significantly and reached a maximum at 7.5 days which was similar to that of the mineralization-related enzymes activities. ACCBP, nacrein and Pif80 were also detected in the mantle culture medium by western blot analysis (Figure 3D, E, F). These results showed that matrix proteins were synthesized intracellularly and secreted by the mantle cells, which allowed the matrix proteins to control crystal polymorph and growth. The function of the matrix proteins found in the cell culture medium was further confirmed using *in vitro* crystallization experiments (Figure 3G, H, I), the results of which suggested that cell-mediated crystals are formed by the regulation of matrix proteins.

When the concentration of Ca^2+^ and Mg^2+^ in the medium was increased, a significant amount of crystals were generated in the 25-day-old mantle cells with Pf-C10M50 (Figure 4E), confirming that the crystals were biogenic and that the mantle cells were responsible for crystal nucleation *in vitro*. The crystals embedded in mantle cells were identified as calcium carbonate based on the higher levels of Ca signal than the surrounding regions of the cell using an EDS analysis (Figure 4G, H), a result that is consistent with the birefringence detected in the mantle cells by polarized light microscopy. Furthermore, the P found in the calcium carbonate is similar to ACC reported by Jacob *et al.* [32]. Indeed, XRD analyses of these minerals confirmed that the polymorph of calcium carbonate that precipitated inside the cell in our cell cultures was ACC (Figure 5), which was also reported for the early veliger stages of *Pinctada fucata* [11]. However, this result is different from calcium carbonate located in the adherent mantle explants and the cell outgrowth area in our previous studies [21]. Amorphous materials are not birefringent under polarized light, to our surprise, ACC in mantle cells can be birefringent in this study. Birefringent areas may act as center of calcification containing different ratios of amorphous to crystalline material depending on the area [10], but the trace of crystalline material cann't detect by us in our studies; another explanation is that ACC-containing vesicles in mantle cells may exhibit birefringence due to its highly organized structure or special organic compounds [37]. The similar statement can also be shown in the literatures like Auzoux-Bordenave *et al.* [38]. ACCBP induces the formation of ACC [28], and compared with Pif80 and nacrein, the expression of ACCBP was comparatively high from day 10 to 25 during cell culture, suggesting that ACCBP was involved in the synthesis of intracellular ACC (Figure 3A). The *de novo* ACC formation by the cells was confirmed by the incorporation of Sr into calcium carbonate (Figure 4F, J). Further work is necessary to determine how ACC transforms into aragonite responsible for shell formation.

The cellular basis of pearl oyster shell formation is consistent with many other biomineralization processes, such as spicule formation. Beniash *et al.* showed the presence of ACC-containing vesicles in the mineralizing cells of sea urchin embryos and suggested that ACC is introduced into the spiculogenic compartment by fusion of the vesicle membrane with the cell membrane of the syncytium [39], thus each spicule forms in a specialized vacuole inside a multicellular vesicle termed the syncytium. However, a cell-mediated shell formation hypothesis that crystal nucleation is intracellular and that crystallogenic cells supply crystals to the mineralization front challenges the classical matrix-mediated hypothesis that shell components are assembled via the self-assembly of materials present in the extrapallial fluid. Based on the results of our present study, we propose that shell formation may involve a mechanism in which ACC is formed in the intracellular vesicles within the mantle cell, is then transported to the extrapallial fluid cavity and subsequently transforms into aragonite via matrix protein regulation. Ultrastructural analyses of the mantle of the gastropod *Haliotis asinina* also support the cell-mediated hypothesis [40]. In our previous studies, some “imprints” of aragonite were found, accompanied with “shaped” aragonite, the “ imprint” is probably the trace of the mantle cell that abuts the inner shell surface [41]. Addadi *et al.* proposed that the mantle cells must be juxtaposed to the mineralizing matrix where and when the shell is being produced [1]. So cells are delivering crystals to the mineralization front then it is likely that those cells will remain attached to the shell. Zhang *et al.* also proposed that oyster shell is not formed simply by matrix proteins but by diverse proteins through complex assembly and modification processes that may involve cells and exosome-like vesicles, which means from the cellular remains due to cellular biomineralization [42],[43]. This study provides cellular evidence for their probable participation in shell formation.

Our research demonstrates the potential of mantle cell culture for studying the biological control of biomineralization in shell formation at the cellular level, which will provide a critical tool to investigate the nucleation and growth of calcium carbonate crystals through the cooperation of mantle cell types in the shell formation process.

## Supporting Information

Table S1 **Primers sequences for the genes used in the real-time PCR analysis.**
(DOCX)
