# Correction: Amorphous Calcium Carbonate Precipitation by Cellular Biomineralization in Mantle Cell Cultures of *Pinctada fucata*

**DOI:** 10.1371/journal.pone.0116034

**Published:** 2014-12-16

**Authors:** The PLOS ONE Staff

The third, fifth, sixth, and seventh author’s names are displayed incorrectly. The correct author list should display as follows: Liang Xiang, Wei Kong, Jing-Tan Su, Jian Liang, Gui-You Zhang, Li-Ping Xie and Rong-Qing Zhang. The correct citation is: Xiang L, Kong W, Su J-T, Liang J, Zhang G-Y, et al. (2014) Amorphous Calcium Carbonate Precipitation by Cellular Biomineralization in Mantle Cell Cultures of *Pinctada fucata*. PLoS ONE 9(11): e113150. doi:10.1371/journal.pone.0113150
