# Supplementary material for: Amorphous Calcium Carbonate Precipitation by Cellular Biomineralization in Mantle Cell Cultures of Pinctada fucata
Source: PLoS One. 2014 Nov 18;9(11):e113150. doi: 10.1371/journal.pone.0113150 (PMC4236139; doi:10.1371/journal.pone.0113150)
Supplement: Table S1 — (DOCX) [file pone.0113150.s001.docx]

**Table S1. Primers sequences for the genes used in the real-time PCR analysis.**

| primer | sequence (5’-3’) |
| --- | --- |
| nacrein-F | GAGCCAGAGGATGGGGAAA |
| nacrein-R | GCCTCCATAGGTGTGAAACGA |
| Pif80-F | TGCTGCCATCACGTGAGTATG |
| Pif80-R | GACTTCCCTTTCTCACACTTCCA |
| ACCBP-F | GACATGGAACAAAGATGGTGGA |
| ACCBP-R | CTGTGGCTGGAATGGTTGG |
| actin-F | CTCCTCACTGAAGCCCCCCTCA |
| actin-R | ATGGCTGGAATAGGGATTCTGG |
